# CXCR4 Antagonism Reduces Cardiac Fibrosis and Improves Cardiac Performance in Dilated Cardiomyopathy

**DOI:** 10.3389/fphar.2019.00117

**Published:** 2019-02-19

**Authors:** Po-Yin Chu, Mandar S. Joshi, Duncan Horlock, Helen Kiriazis, David M. Kaye

**Affiliations:** ^1^Heart Failure Research Group, Baker Heart and Diabetes Institute, Melbourne, VIC, Australia; ^2^Experimental Cardiology Laboratory, Baker Heart and Diabetes Institute, Melbourne, VIC, Australia; ^3^Department of Medicine, Monash University, Melbourne, VIC, Australia

**Keywords:** cardiomyopathy, chemokine receptor, CXCR4 antagonism, heart failure, cardiac fibrosis, cardiac performance, receptor antagonist

## Abstract

**Background:** Myocardial fibrosis is a key pathologic finding in the failing heart and is implicated as a cause of increased ventricular stiffness and susceptibility to ventricular arrhythmia. Neurohormonal mediators such as aldosterone and angiotensin II are known to cause fibrosis in experimental models, however, clinical evidence for the reversal of fibrosis with relevant antagonists is limited. Recent studies suggest that inflammatory mediators may contribute to fibrosis. In dilated cardiomyopathy the mechanism for myocardial fibrosis is unclear and its implications on systolic function are not known.

**Methods and Results:** We studied the effect of a highly selective antagonist of SDF-1/CXCR4 signaling, AMD3100, on the development of cardiac fibrosis and cardiac function in mice with dilated cardiomyopathy due to cardiac-specific transgenic overexpression of the stress-kinase, Mst1. AMD3100 significantly attenuated the progression of myocardial fibrosis and this was accompanied by significant improvements in diastolic and systolic performance as evaluated in isolated Langendorff perfused hearts. AMD3100 reduced BNP mRNA expression but did not alter the expression of Ca^2+^ handling genes. CXCR4 antagonism also reduced the abundance of splenic CD4^+^ T cells.

**Conclusion:** This study demonstrates that CXCR4 pathway contributes to pathogenesis of cardiac fibrosis in dilated cardiomyopathy, and it represents a new potential therapeutic target in heart failure. The data also demonstrate that anti-fibrotic strategies can improve systolic performance.

## Introduction

Cardiac remodeling, incorporating structural, functional and molecular alterations are fundamental findings in the failing heart (HF). More specifically individual patterns of remodeling, together with extra-cardiac abnormalities, contribute to the particular clinical phenotype. Expansion of the extracellular matrix with deposition of proteins including collagen type I and III, is a common finding in the large majority of HF. The extent of myocardial fibrosis has been demonstrated to be an important contributor to ventricular stiffness and arrhythmogenesis (Burchfield et al., 2013). Arising from the development of non-invasive tools to assess the extent of cardiac fibrosis using cardiac magnetic resonance imaging (Moon et al., 2006; Iles et al., 2008; Puntmann et al., 2016), it is now evident that the extent of myocardial fibrosis is strongly associated with outcome in HF patients with reduced ejection fraction (HFREF) and those with preserved ejection fraction (HFPEF) (Iles et al., 2011; Mascherbauer et al., 2013). As such, a continuing clinical need exists for the development of effective anti-fibrotic therapies.

The pathogenesis of cardiac fibrosis is complex. Although angiotensin II and aldosterone have been ascribed a lead role in the pathogenesis of cardiac fibrosis, the clinical effectiveness of antagonists at substantially reversing cardiac fibrosis is variable and limited (Brilla et al., 2003; Kitzman et al., 2010). Recent data suggests that other pathways may also be important contributors to the accumulation of cardiac fibrosis; these include oxidative stress mediators, inflammatory cytokines such as TGFβ, and infiltrating macrophages. All of these appear to play an important role in modulating and stimulating fibroblast proliferation and differentiation. Emerging data demonstrates that manipulation of such targets can modify cardiac fibrosis development in selected experimental models including angiotensin II infusion and acute pressure overload elicited by transverse aortic constriction (Kain et al., 2016). Notwithstanding their role in cardiac fibrosis, the role of more targeted interventions such as macrophage depletion or TGFβ inhibition in dilated cardiomyopathy remains unknown.

In the present study we addressed the hypothesis that selective CXCR4 antagonism might prevent the development of myocardial fibrosis in an experimental model of dilated cardiomyopathy. This notion is based upon our finding, and by others, that levels stromal derived factor-1 (SDF-1), which binds to CXCR4, are elevated in experimental and human heart failure (Chu et al., 2010; Subramanian et al., 2014; Baerts et al., 2015). Recent studies have demonstrated that SDF-1 activates cardiac fibroblasts and collagen production (Jackson et al., 2017, 2018). Furthermore, it has been demonstrated that activation of the SDF1/CXCR4 axis can be pro-fibrotic and pro-inflammatory in experimental models of lung injury and hypertension (Strieter et al., 2007; Chu et al., 2011).

## Materials and Methods

### Animals

All experimental protocols were approved by the Alfred Medical Research and Education Precinct Animal Experimentation Ethics Committee under the guidelines of the National Medical and Health Research Council of Australia.

To investigate the impact of CXCR4 antagonism as a preventive strategy in dilated cardiomyopathy, thirty-two male mice age 6 weeks (16 Mst1 mice and 16 wild-type C57BL/6 mice) were used in our study. The Mst1 transgenic mice have been described in detail previously (Yamamoto et al., 2003). In brief, Mst1 transgenic mice were generated on a C57BL/6 background, and the stress kinase, Mst1, was overexpressed in a cardiac-specific manner using the α-myosin heavy chain promoter. Animals develop a dilated cardiomyopathy phenotype which is established by 10 weeks of age. Animals were randomly allocated to receive the selective CXCR4 antagonist AMD3100 (6 mg/kg per day, Tocris Bioscience, Bristol, United Kingdom) or vehicle (H_2_O), delivered by a mini-osmotic pump (Alzet, Model 2004). The dose selected was based upon our previous work using another CXCR4 inhibitor, AMD3465 (Chu et al., 2011). The study included the following 4 intervention groups: control (C57BL/6 mice, vehicle mini-osmotic pump, *n* = 8); control+AMD3100 (C57BL/6 mice, AMD3100 mini-osmotic pump, *n* = 8); Mst1 (Mst1 mice, vehicle mini-osmotic pump, *n* = 8); and Mst1+AMD3100 (Mst1 mice, AMD3100 mini-osmotic pump, *n* = 8). All animals received a standard diet and were followed by weekly measurements of weight and monitored for a period of 12 weeks. This duration was selected based upon the hypothesis that antagonism of SDF-1/CXCR4 signaling would abrogate the development of the cardiac phenotype in Mst-1 transgenic mice, which is typically advanced by 18 weeks.

### Functional Measurements

On the day before study completion, echocardiographic imaging of the left ventricle was performed under isoflurane anesthesia, using a PHILIPS IE33 ultrasound machine (Royal Philips Electronics, Amsterdam, The Netherlands) with a 15-MHz linear transducer. Off-line image analysis was performed in a blinded fashion. Before termination, arterial blood pressure was measured, using a 1.4 F microtipped transducer catheter (Millar) inserted through the carotid artery.

### Gross Morphometry and Histological Analysis

Animals were killed at the end of the experiments by deep anesthesia and the heart, kidney and lung were rapidly excised, washed and weighed. Hearts were immersed in saline on ice before fixing half of the left ventricle in 10% formalin for paraffin sectioning and the remaining half was snap-frozen in liquid nitrogen for molecular biology.

Ventricular tissue was prepared for paraffin sectioning. Four-micron paraffin sections were stained with Masson’s Trichrome to evaluate the distribution and localization of collagen. The extent of fibrosis was measured in each of ten randomly chosen fields per animal in perivascular and interstitial areas with ImagePro Plus software (Adept Electronic Solutions Pty Ltd., Moorabbin, VIC, Australia) using an Olympus BH2 microscope with results expressed as a percentage of blue area in each screen at a magnification of 400×. Perivascular and interstitial collagen volume fraction of the Masson’s Trichrome stained tissue were measured separately. All collagen surrounding an intramyocardial coronary artery was considered as perivascular collagen. Vessels that were located in scars were excluded from the analysis. Image analysis was performed by an investigator who was unaware of the assigned treatment group.

### Isolated Left Ventricular Performance

Left ventricular performance was investigated in isolated Langendorff perfused hearts. The investigator performing the studies was unaware as to group allocation. Mice were anesthetized with an intraperitoneal injection of sodium pentobarbitone (50 mg/kg), a thoracotomy was performed and hearts rapidly excised into ice-cold perfusion fluid. The aorta was cannulated on a shortened blunt 21 gauge needle and perfusion initiated at a constant pressure of 80 mmHg on the apparatus. Thebesian fluid accumulation into the left ventricle was vented via a polyethylene drain through the apex of the heart, and a fluid-filled balloon constructed from polyvinyl chloride film was introduced into the left ventricle through an incision in the atrial appendage. The ventricular balloon was connected via fluid-filled tubing to a pressure transducer (ADInstruments, Castle Hill, NSW, Australia) for continuous assessment of ventricular performance. The balloon was initially inflated to yield a left ventricular end-diastolic pressure of 5 mmHg during the 15 min of stabilization. Hearts were immersed in warmed perfusate in a jacketed bath maintained at 37 C, and perfusate delivered to the coronary circulation was maintained at the same temperature. The perfusate was modified Krebs-Henseleit solution containing (in mM): NaCl, 120; NaHCO_3_, 25; KCl, 4.7; CaCl_2_, 2.5; MgCl_2_ 1.2; KH_2_PO_4_ 1.2, D-glucose, 15; and EDTA, 0.5. Perfusion fluid was maintained at 37°C and bubbled with a mix of 95% O_2_/5% CO_2_ at 37°C to provide a pH of 7.4. Organ bath and perfusate temperatures were continuously monitored using a three channel Physitemp TH-8 digital thermometer (Physitemp Instruments Inc., Clifton, NJ, United States). Ventricular pressure signals were recorded on a four-channel MacLab data acquisition system (ADInstruments). Left ventricular pressure was digitally processed to yield systolic, end-diastolic and developed (LVDP) pressures, heart rate, and the peak positive and negative differentials of pressure change with time (+dP/dt and -dP/dt, respectively). The capacity of the left ventricular to respond to increasing preload was subsequently determined during stepwise increases in the left ventricular diastolic (balloon) pressure.

### Molecular Biology

Real-time PCR was performed to determine collagen types I and III, brain natriuretic peptide (BNP), sarcoplasmic reticulum ATPase type 2a (SERCA2a), phospholamban (PLN), ryanodine receptor, sodium calcium exchanger (NCX) and SDF-1α mRNA expression in the heart. Total RNA was isolated using the TRIzol (Invitrogen) purification system and reverse transcribed using TaqMan reagents (Applied Biosystems). Real-time PCR (ABI Prism 7300 sequence detection system, Applied Biosystems) was conducted using a 50-ng template. The primers sequences for real-time PCR included collagen I: (fwd) 5^′^-GGAGATGATGGGGAAGCTG-3^′^, (rev) 5^′^- AATCCACGAGCACCCTGA-3^′^; collagen III (fwd) 5^′^-GGAATGGAGCAAGACAGTCTTTG-3^′^, (rev) 5^′^-TGCGATATCTATGATGGGTAGTCTCA-3^′^; PLN (fwd) 5^′^-ACAGAAAACTGCCCAGCTAAGC-3^′^; (rev) 5^′^-CGT CACAGTGCAGAGCATGA-3^′^; Ryr2 (fwd) 5^′^-CCAAGCACGGCAGTCACA-3^′^; (rev) 5^′^-GCTCAACCCACTTAT AGTAAGGCACTA-3^′^; SERCA2a (fwd) 5^′^-TCTGGAGTTTTCACGGGATAGAA; (rev) 5^′^-TCCGGCTTGGCT TGTTTG; NCX (fwd) 5^′^-GGATTCAAGCTA CTCGCCTGAT-3^′^; (rev) 5^′^-GGTCGTTTTCAGCCATTTCC-3^′^; SDF-1 (fwd) 5^′^-AACCCACCATGCTCATCATTC-3^′^, (rev) 5^′^-TTTCAGGGTCATGGAGACAGTCT- 3^′^ and GAPDH (fwd: 5^′^-ACAGCAACTCCCACTCTTCC- 3^′^, rev: 5^′^-CCTCTCTTGCTCAGTGTCC-3^′^). Expression values were determined by calculating the ΔΔCt value for each reaction, and data were expressed as fold difference compared with control.

### Flow Cytometry

To investigate the potential role of T cell activation in the pathophysiology of the Mst1 cardiac phenotype and the effects of CXCR4 antagonism, we performed flow cytometric analysis of splenic homogenates. Splenic tissue was homogenized and cells were pelleted and then suspended in FACS buffer. Cells were subsequently stained for CD4, CD25, and FoxP3 (eBioscience) according to the manufacturer’s instructions, followed by flow cytometric analysis (BD FACS Canto LSRII flow cytometer).

### Statistical Analyses

The procedures were carried out using the Statistical Package for the Social Sciences (SPSS) software (SPSS 17.0 for Windows, SPSS, Chicago, IL, United States). Values are presented as the mean ± SEM. As appropriate, between group comparisons were performed using Student *t*-test for normally distributed data. Comparisons where relevant between 3 or more groups were performed with ANOVA with Tukey’s *post hoc* analysis. A *p*-value of <0.05 was considered to be significant.

## Results

### Effects of CXCR4 on Cardiac Morphometry and Fibrosis

The primary objective of the study was to investigate the effect of the CXCR4 antagonist, AMD3100, on ventricular structure and function in mice with dilated cardiomyopathy. Gross morphometry demonstrated a lower body weight in Mst1 compared to controls (*p* < 0.05) and, interestingly, this appeared to be abrogated in Mst1 treated with AMD3100 (*p* < 0.05, Table 1). In keeping with the original description, Mst1 mice did not demonstrate evidence of compensatory cardiac hypertrophy as evidenced by a lack of increase in the heart to body weight ratio (Table 1). Consistent with the cardiomyopathy phenotype, Mst1 mice displayed evidence of lung congestion (Table 1) as reflected by an increase in lung weight and lung weight indexed to body weight. In Mst1 mice treated with AMD3100 there was no change in total lung weight, however, the body weight indexed lung weight was lower as shown in Table 1 (*p* < 0.05).

**Table 1:** Gross morphometry.

	Control	AMD3100	Mst-1	Mst-1 +AMD3100
Body weight (gm)	35.6 ± 0.6	35.8 ± 1.1	31.5 ± 1.0*	35.7 ± 0.6^†^
Heart weight (mg)	170 ± 10	161 ± 4	165 ± 4	172 ± 8
Heart:BW	4.8 ± 0.4	4.7 ± 0.2	5.4 ± 0.1	4.8 ± 0.2
Lung weight (mg)	197 ± 7	208 ± 1	218 ± 4*	215 ± 7
Lung:BW	5.6 ± 0.2	5.9 ± 0.4	6.9 ± 0.3*	6.0 ± 0.3^†^
Kidney weight (mg)	250 ± 12	245 ± 9	221 ± 5	215 ± 6
Kidney:BW	7.1 ± 0.3	7.1 ± 0.3	6.9 ± 0.3	6.2 ± 0.1


*Data are mean ± SEM (n = 5–8); BW, body weight; ^∗^p < 0.05 vs. control; ^†^p < 0.05 vs. Mst-1.*

**FIGURE 1:**
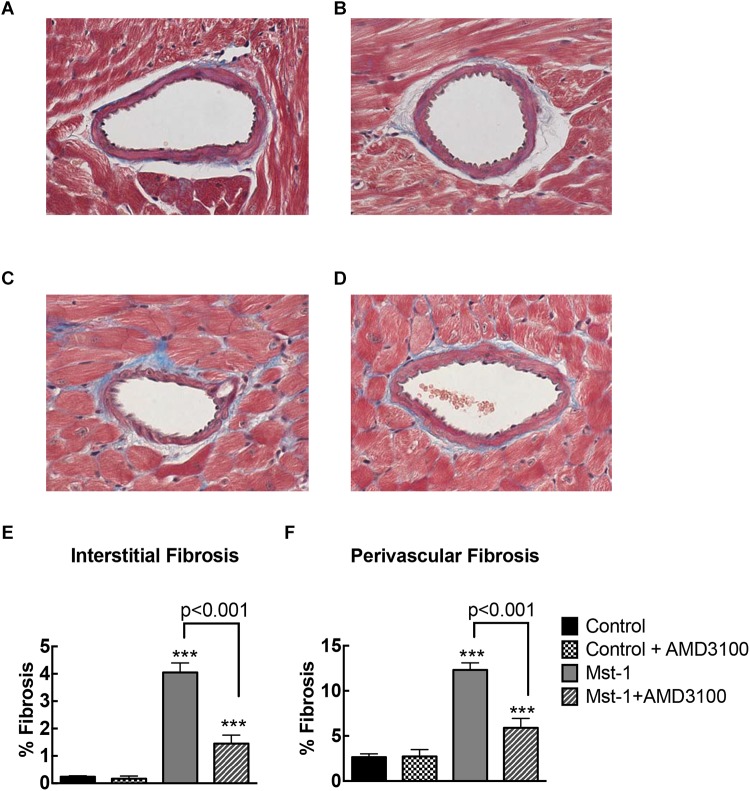
(**A–D**) Representative photomicrographs of Masson’s stained myocardial sections from control **(A)**, control+AMD3100 **(B)**, Mst-1 **(C)**, and Mst-1+AMD3100 **(D)**. **(E,F)** Bar graphs represent perivascular and interstitial collagen volume fraction. ^∗∗∗^*p* < 0.001 vs. control.

Quantitative analysis of myocardial sections demonstrated significant accumulation of perivascular fibrosis (control vs. Mst1: 2.7 ± 0.2 vs. 12.3 ± 0.3%, *p* < 0.001) and interstitial fibrosis (control vs. Mst1: 0.2 ± 0.1 vs. 4.1 ± 0.1%, *p* < 0.001) in cardiomyopathy mice as represented in Figure 1. The accumulation of both perivascular fibrosis and interstitial fibrosis were markedly attenuated in Mst1 mice treated with AMD3100 (both *p* < 0.001 vs. Mst1), whilst AMD3100 alone was without effect in control mice.

### Effect of CXCR4 Antagonism on Cardiac Function

As shown in Table 2, cardiomyopathic mice exhibited significant left ventricular dilatation in conjunction with significantly reduced systolic performance, compared to wild type mice as assessed by echocardiography. Furthermore, cardiac specific Mst1 overexpression was accompanied by left ventricular dilatation and reduced wall thickness. CXCR4 antagonist administration was associated a significant attenuation in ventricular wall thinning, however, fractional shortening was not changed (Table 2). AMD3100 alone was without effect on any echocardiographic index of left ventricular size or function. Consistent with significantly reduced contractile function, hemodynamic evaluation showed that Mst1 mice had a lower systolic blood pressure (control vs. Mst 1: 92 ± 3 vs. 75 ± 2 mmHg, *p* < 0.01) and an increased left ventricular end-diastolic pressure compared to wild type mice (Control vs. Mst 1: 5.4 ± 0.3 vs. 11.3 ± 0.9 mmHg, *p* < 0.05). AMD3100 did not alter systolic blood pressure in control mice (95 ± 2 mmHg) or in Mst-1 mice (70 ± 5 mmHg). LVEDP was also unaltered by ADM3100 in control (6.6 ± 1.3 mmHg) or Mst-1 mice (12.0 ± 2.3 mmHg).

**Table 2:** Echocardiographic parameters.

	Sham Control	Control +AMD3100	Mst-1	Mst- 1+AMD3100
IVSd, mm	0.73 ± 0.02	0.70 ± 0.01	0.63 ± 0.01^∗^	0.63 ± 0.01^∗^
LVIDd, mm	4.13 ± 0.01	3.97 ± 0.03	4.37 ± 0.06^∗^	4.40 ± 0.05^∗^
LVPWd, mm	0.72 ± 0.01	0.75 ± 0.03	0.67 ± 0.01^∗^	0.82 ± 0.02^∗†^
IVSs, mm	1.27 ± 0.04	1.20 ± 0.03	0.76 ± 0.02^∗^	0.99 ± 0.02^∗^†
LVIDs, mm	2.69 ± 0.08	2.60 ± 0.07	3.37 ± 0.08^∗^	3.39 ± 0.03^∗^
LVPWs, mm	1.09 ± 0.03	1.15 ± 0.02	0.78 ± 0.03^∗^	1.03 ± 0.01†
FS (%)	35 ± 2	35 ± 2	23 ± 2^∗^	23 ± 1^∗^


*IVSd, interventricular septal diastolic thickness; LVIDd, left ventricular internal diameter at end diastole; LVPWd, left ventricular posterior wall thickness at end diastole; IVSs, interventricular septal systolic thickness; LVIDs, left ventricular internal diameter at end systole; LVPWs, left ventricular posterior wall thickness at end systole; FS, fractional shortening. Values are means ± SE (n = 6); ^∗^P < 0.05 vs. Sham Control; ^†^P < 0.05 AMD3100 vs. Mst-1.*

To investigate cardiac function in greater detail and in the absence of the potentially confounding influence of anesthesia, we next evaluated left ventricular performance using isolated Langendorff perfused hearts (*n* = 4 per group). As shown in Table 3, both peak positive and negative dP/dt were significantly reduced in Mst1 mice compared to controls. Administration of AMD3100 to Mst1 mice was accompanied a significant improvement in both peak positive and negative dP/dt toward normal (Table 3).

**Table 3:** Contractile function in isolated perfused hearts.

Groups	Basal LV Balloon pressure (5 mmHg)		LV Balloon pressure (10 mmHg)		LV Balloon pressure (20 mmHg)	
	+dP/dt (mmHg/s)	-dP/dt (mmHg/s)	+dP/dt (mmHg/s)	-dP/dt (mmHg/s)	+dP/dt (mmHg/s)	-dP/dt (mmHg/s)
Control	4717 ± 216.3	-3305.0 ± 143.7	4693.7 ± 238.9	-3336.7 ± 113	4601 ± 320.9	-3357.2 ± 172.6
Mst1	3367.6 ± 113.9^∗^	-2670 ± 137.1^∗^	3492.9 ± 104^∗^	-2511.1 ± 155.5	3258.8 ± 111.7^∗^	-2344.8 ± 129.3^∗^
Mst1+AMD3100	4145.2 ± 306.8^†^	-3217.3 ± 140.1^†^	4188 ± 181.6^†^	-3141.6 ± 99.2^†^	3677.6 ± 233.3	-2966.6 ± 111.1^†^


*Data are mean ± SEM (n = 7–8 per group); ^∗^p < 0.05 vs. C57BL6; ^†^p < 0.05 vs. Mst1.*

### Myocardial Gene Expression

**FIGURE 2:**
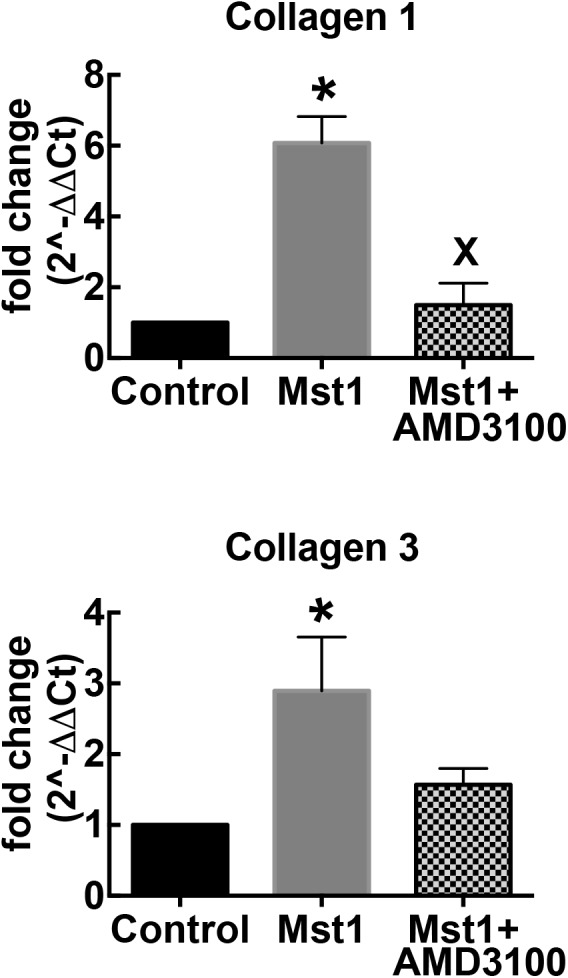
Bar graphs representing relative collagen I and collagen III mRNA expression. ^∗^*p* < 0.05 vs. control; ^x^*p* < 0.05 vs. Mst-1.

To complement the histological studies on myocardial fibrosis, we evaluated the expression of collagen types I and III mRNA by RT-PCR. Consistent with the histological data, collagen I and III gene expression in the failing heart was significantly increased in mice with cardiomyopathy (Figure 2). The administration of AMD3100 significantly reduced collagen type I mRNA, and there was a non-significant trend toward collagen type III mRNA abundance. Treatment with the CXCR4 antagonist alone in wild type mice was without effect.

**FIGURE 3:**
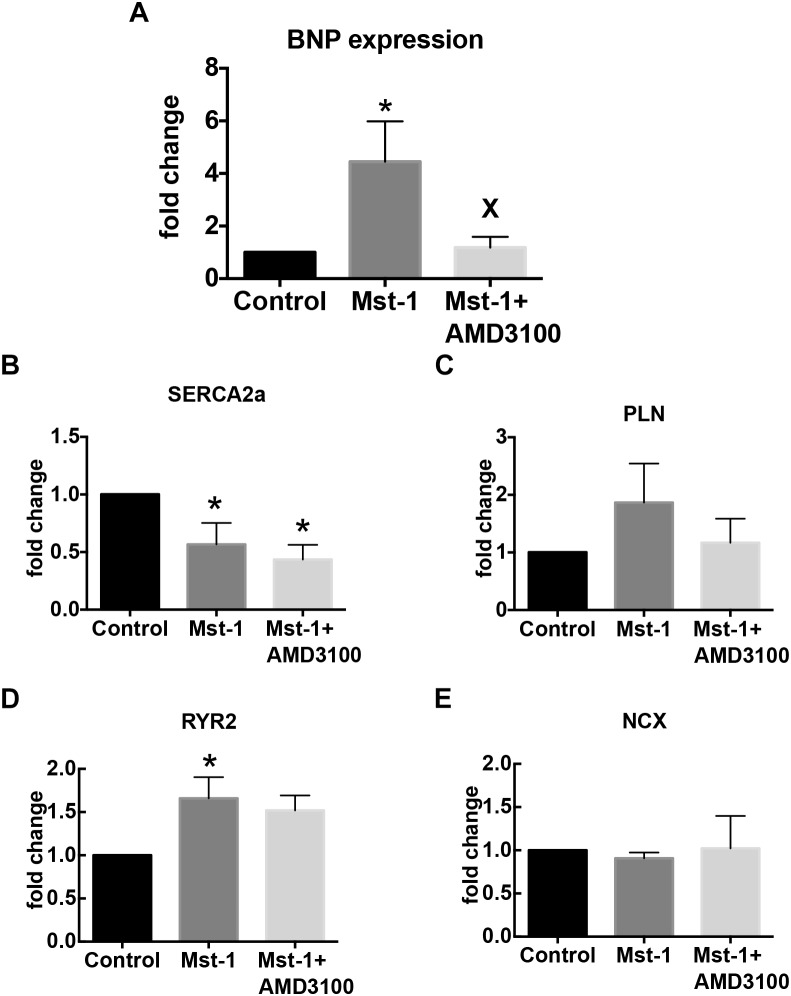
Bar graphs representing **(A)** BNP, **(B)** SERCA, **(C)** phospholamban, **(D)** ryanodine receptor, and **(E)** sodium-calcium exchanger mRNA expression. ^∗^*p* < 0.05 vs. control; X*p* < 0.05 vs. Mst-1.

To further elucidate the mechanism of the actions of CXCR4 antagonism on the failing myocardium we first characterized SDF-1 mRNA. Consistent with previous studies we demonstrated a 35 ± 8% increase in SDF-1 mRNA in Mst1 ventricular myocardium compared to wild type control (*n* = 3 per group, *p* < 0.05) (Chu et al., 2010). SDF-1 mRNA levels were not significantly influenced by AMD3100. In conjunction, we investigated the effects of AMD3100 administration on the levels of expression of mRNA for brain natriuretic peptide (BNP), sarcoplasmic reticulum ATPase 2a (SERCA), the ryanodine receptor, Ryr2, and PLN. As shown in Figure 3, AMD3100 administration was accompanied by significant a reduction in the expression of BNP mRNA in Mst1 mice from the elevated levels observed in untreated Mst-1 mice. Expression levels of SERCA were significantly reduced in Mst1 mice and were not altered by AMD3100 (Figure 3). RyR mRNA expression was increased significantly in Mst-1 mice, but remained unaltered in AMD3100 treated Mst-1 mice. PLN and NCX mRNA expression was not significantly altered in Mst-1 mice or in Mst-1 mice treated with AMD3100 (Figure 3).

### Immunomodulatory Effects of CXCR4 Antagonism in Mst1 Mice

To address the possibility that the anti-fibrotic action of CXCR4 antagonism is mediated via an immunomodulatory action we performed FACS analysis of splenic homogenates. CD4 positive cells represented 16.9 ± 1.3% of splenic cells in and this was modestly reduced in Mst-1 mice (12.4 ± 1.2%, *p* = 0.05). In Mst-1 mice, treatment with the CXCR4 antagonist reduced the frequency of CD4+ cells to 7.2 ± 1.2% (*p* < 0.05). Splenic regulatory T cell abundance in Mst-1 mice was unaffected by AMD3100, as reflected by the relative proportions of Foxp3^+^CD4^+^ cells (Mst1 vs. Mst-1 +AMD3100: 10.3 ± 1.2 vs. 9.4 ± 1.9%, *p* = ns) or CD25^+^CD4^+^ cells (Mst1 vs. Mst1+AMD: 8.8 ± 0.9 vs. 8.9 ± 1.0%, *p* = ns).

## Discussion

Our findings demonstrate that activation of the SDF-1/CXCR4 axis plays an important role in the structural and functional remodeling of the failing heart. In mice with experimental dilated cardiomyopathy we showed that administration of AMD3100, a highly selective CXCR4 antagonist, was associated with a marked reduction in perivascular and interstitial fibrosis, supported by a reduction in the expression of collagen I mRNA. These findings are consistent with reports of increased heart failure in patients treated with dipeptidyl peptidase-4 inhibitors, given that these drugs augment SDF-1 levels by inhibiting degradation (Packer, 2018). Diffuse cardiac fibrosis is a structural hallmark of ventricular remodeling, contributing to an increase in ventricular stiffness (Westermann et al., 2008; Ellims et al., 2014). Pathophysiologically, increased myocardial stiffness causes an increase in ventricular filling pressures which in turn contributes to the symptoms of HF via pulmonary congestion (Maeder et al., 2010; Houstis and Lewis, 2014). We found that lung weight and indexed lung weight was elevated in Mst-1 mice consistent with pulmonary congestion, and AMD3100 reduced the indexed lung weight, consistent a reduction in pulmonary congestion. However, there was no change in lung weight *per se* and without an assessment of lung water the interpretation of the change in indexed lung weight remains speculative.

Remodeling of the myocardial interstitium by cardiac fibroblasts, and other cells, represents the net balance between the secretion of matricellular proteins and their degradation by proteolytic enzymes including the matrix metalloproteinases (Frangogiannis, 2012). The origin of secretory cardiac fibroblasts, is complex and potentially includes contributions from resident cardiac fibroblasts, recruited circulating fibrocytes, vascular pericytes, endothelial to mesenchymal transition, and pericardial cells (Krenning et al., 2010; Furtado et al., 2014). Activation of the renin-angiotensin-aldosterone system has been proposed to be a principal driver of cardiac fibrosis. Whilst, the use of angiotensin converting enzyme inhibitors, angiotensin receptor antagonists and aldosterone antagonists provides clear benefit in HF, evidence for an anti-fibrotic action in patients is limited (Brilla et al., 2003; Kitzman et al., 2010). In the current study, Mst1 mice had evidence of ventricular dilatation and reduced systolic function which was not altered by AMD3100. We observed some evidence for attenuation of ventricular wall thinning although this was not accompanied by a change in fractional shortening.

Immune activation, potentially triggered by elevated oxidative stress and cardiomyocyte damage is also an important contributor to cardiac fibrosis. Amongst the mediators of fibrosis, recruited macrophages play an important role via secretion of TGFβ, together with other cytokines, which stimulate fibroblast proliferation and differentiation, together leading to the release of chemotactic, and other pro-inflammatory signals (Glezeva and Baugh, 2014). We previously demonstrated an increase in the expression of stromal derived factor-1 (SDF-1) in the setting of experimental and clinical heart failure (Chu et al., 2010), however, it remained unclear as to whether this was a mediator of fibrosis. Conceptually, SDF-1 acting via its cognate receptor, CXCR4 may increase fibrosis by stimulating fibroblast proliferation and recruitment or alternately may stimulate inflammation via T cell recruitment (Bryant et al., 2012). We also previously showed that selective CXCR4 antagonism attenuated the development of cardiac fibrosis in experimental hypertension (Chu et al., 2011).

Recent studies performed in pressure overload and mineralocorticoid excess models of cardiac hypertrophy and fibrosis increasingly suggest a key role for activation of inflammatory cells and cytokines. In particular, CD4^+^ T cells have been strongly implicated as a driver of cardiac fibrosis (Laroumanie et al., 2014; Nevers et al., 2015), likely through pro-inflammatory Th1 and Th17 subsets and their respective cytokine mediators including IL-6, IL1β, TNF, and IL17 (Madhur et al., 2010; Amador et al., 2014) which may cause fibrosis by activation of resident cardiac fibroblasts. Consistent with this immunological paradigm, we found that CXCR4 antagonism led to a significant reduction in the abundance of splenic CD4^+^ T cells. This finding is consistent with previous studies showing that SDF-1 activates T cells (Nanki and Lipsky, 2001). Furthermore, recent studies showing that T cell depletion attenuated the development of cardiac fibrosis and the progression to heart failure in the pressure overload model of hypertrophy (Nevers et al., 2015). Further to the abundance of CD4+ cells, recent data also indicate that the balance between pro-inflammatory T cells and anti-inflammatory T cells (particularly regulatory T cells) is also important in the development of cardiac fibrosis (Kanellakis et al., 2011). In the present study we did not find evidence of increased numbers of splenic regulatory T cells. In addition to the immunomodulatory action of CXCR4 inhibition, it has also been demonstrated that CXCR4 inhibition can reduce the expression of MMP-9 (Sekula et al., 2014), although this was not measured in the current study. This observation is potentially relevant, given the well documented relationship between increased MMP-9 expression and matrix remodeling in heart failure (Kelly et al., 2007; Spinale, 2007).

The SDF-1/CXCR4 axis has previously been studied in the setting of myocardial infarction (MI) and ischemia reperfusion injury. Elevated SDF-1 expression occurs early after MI, contributing to the recruitment and retention of endothelial progenitor cells arising from the bone marrow (Aiuti et al., 1997; Shintani et al., 2001). Within bone marrow, the SDF-1/CXCR4 axis plays a role in the retention of progenitor cells (Peled et al., 1999), and its inhibition has been shown to promote the mobilization of stem cells. Within this framework, single dose administration of CXCR4 antagonists have been shown to augment recovery from ischemic injury (Jujo et al., 2013; Hsu et al., 2015). CXCR4 antagonism has been shown to promote the mobilization of endothelial precursor cells and mesenchymal stem cells with improvements in cardiac performance potentially mediated by enhanced angiogenesis, reduced apoptosis or reduced inflammation (Jujo et al., 2013; Hsu et al., 2015).

We found that left ventricular systolic function, assessed by dP/dt, in isolated perfused hearts was improved in AMD3100 treated Mst-1 mice compared to Mst-1 mice alone. However, this was not mirrored by any changes in systolic function as assessed echocardiographically or via cardiac catheterisation. It is possible that the effects of general anesthesia may have masked the presence of functional differences in intact animals. We did not assess the end-systolic pressure volume relationship, a load insensitive measure of contractile performance. Our study is consistent with several imaging studies which report an inverse correlation between the extent of myocardial fibrosis and indices systolic performance, although this remains controversial (Donekal et al., 2014; Jacquier et al., 2014; Tandon et al., 2015). Mechanistically it has been proposed this observation could be explained by several pathophysiologic mechanisms. These include the combined effects of cardiomyocyte loss together with replacement fibrosis; the effects of impaired coronary flow reserve in the context of perivascular fibrosis; or increased contractile afterload in the setting of interstitial fibrosis. Interestingly it has also been shown that myofibroblasts may signal via paracrine mechanisms to cardiomyocytes, to reduce Ca^2+^ handling and contractility (LaFramboise et al., 2007; Vasquez et al., 2010; Cartledge et al., 2015). In the current study we did not observe any change in the expression of SERCA2a, phospholamban, ryanodine receptor or the sodium calcium exchanger, which are key regulators of calcium handling. Similarly, there was no evidence of altered ventricular mass which would argue against a potential anti-apoptotic action. We did not directly assess the extent of cardiomyocyte apoptosis in this study. Apoptosis, driven by transgenic overexpression of Mst-1 has been shown to be elevated in Mst-1 mice (Yamamoto et al., 2003), and accordingly would be unlikely to be modified by pharmacologic intervention. Given that we did not observe any effect of AMD3100 on cardiac structure or function in control mice, we did evaluate the effects of AMD3100 alone on myocardial gene expression or ventricular function in isolated perfused hearts.

Taken together, the present study shows, for the first time, that chronic CXCR4 antagonism can exert an anti-fibrotic action which is accompanied by evidence of improved cardiac performance in non-ischemic, dilated cardiomyopathy. Further studies are warranted to examine the potential utility of manipulation of the SDF-1/CXCR4 axis as a clinical therapeutic strategy in heart failure.

## Author Contributions

DK and P-YC designed the study. P-YC, HK, MJ and DH conducted the study. All authors contributed to analysis and manuscript preparation. Study funding was acquired by DK.

## Conflict of Interest Statement

The authors declare that the research was conducted in the absence of any commercial or financial relationships that could be construed as a potential conflict of interest.
